# Contradictory Results in Microbiome Science Exemplified by Recent *Drosophila* Research

**DOI:** 10.1128/mBio.01758-18

**Published:** 2018-10-02

**Authors:** Angela E. Douglas

**Affiliations:** a Department of Entomology, Cornell University, Ithaca, New York, USA; b Department of Molecular Biology and Genetics, Cornell University, Ithaca, New York, USA

**Keywords:** *Lactobacillus plantarum*, aging, life span, microbiome, probiotics

## Abstract

The bacterium Lactobacillus plantarum is prevalent in animal guts and is widely regarded as beneficial and probiotic. D. Fast et al. (mBio 9:e01114-18, 2018, https://doi.org/10.1128/mBio.01114-18) make the surprising discovery that L. plantarum reduces the life span of Drosophila melanogaster and link this effect with the loss and weakened proliferation of stem cells in the *Drosophila* gut. These results are apparently at odds with published evidence for beneficial effects of L. plantarum, especially promoting high developmental rates and stimulating stem cell proliferation in young *Drosophila*. The among-study discrepancies highlight the context dependence of many effects of gut microbes on host health, likely influenced by host age and genotype, variation among bacterial strains, and diet. The diversity of results offers an opportunity to elucidate a fundamental mechanism(s) and the circumstances that dictate whether gut bacteria have positive or negative effects on host health. These studies also reinforce the value of *Drosophila* as an emerging model system for probiotic science.

## Abstract (precis)

The bacterium Lactobacillus plantarum is prevalent in animal guts and is widely regarded as beneficial and probiotic. D. Fast et al. (mBio 9:e01114-18, 2018, https://doi.org/10.1128/mBio.01114-18) make the surprising discovery that L. plantarum reduces the life span of Drosophila melanogaster and link this effect with the loss and weakened proliferation of stem cells in the *Drosophila* gut.

## COMMENTARY

The default expectation is that the gut microbiota in a healthy animal makes a positive contribution to host health. Any microorganisms that are deleterious to the host are classified differently—as pathogens. And yet, we know that this simple dichotomy is an oversimplification, with ample evidence that the impact of many microorganisms on the host can be context dependent, varying with the genotype and physiological condition of the host, the composition and activity of other microbial taxa in the gut, and many other factors. This complexity has led to a pressing problem in microbiome science: disconcerting inconsistencies between different studies addressing a single topic and difficulties in making reliable predictions about the impact of gut microorganisms on host health.

The article by Fast et al. (1) provides a vivid illustration of the complexities of gut microbe-host associations and how unexpected results have the potential to lead to a greater understanding of the diversity of interactions in these systems.

The focus of Fast et al. (1) is the gut microbiota of the fruit fly Drosophila melanogaster. *Drosophila* is an excellent system for fundamental discovery in microbiome science, combining the superb genetic and genomic resources of this model insect with a highly tractable microbiology. Indeed, the starting point of the study by Fast et al. was to make use of routine methods to eliminate the microbiota from *Drosophila*, yielding axenic (or germfree) flies, and to synthesize associations with standardized microbiota. Specifically, Fast et al. (1) generated monoassociations with each of the dominant bacteria in their *Drosophila* cultures, including Lactobacillus plantarum (*Firmicutes*) and Acetobacter pasteurianus (*Alphaproteobacteria*).

The traits of the *Drosophila* strains associated with these single bacterial taxa led to the first of two unexpected results in this study: that the flies bearing L. plantarum, but not A. pasteurianus, died prematurely relative to axenic *Drosophila*. Why is this result unexpected? The reason is that microbiologists, including the research community studying *Drosophila* microbiomes, consider L. plantarum to be a beneficial member of the gut microbiota. Representatives of this species are used very widely as a probiotic for human consumption and have documented positive effects on the growth rates of both juvenile mice and larval *Drosophila* raised on low-nutrient diets (2–4).

Attention to two issues may resolve the poor correspondence between the results of Fast et al. (1) and previous studies: among-strain variation in the effect of L. plantarum on *Drosophila* performance and variation in the impact of L. plantarum on different indices of host performance. Interestingly, Fast et al. (1) obtained shortened *Drosophila* life span for all of the three L. plantarum strains tested. However, it would be premature to conclude that this effect is a species-level trait of L. plantarum, because other studies using different L. plantarum strains have revealed significant variation in effects on both *Drosophila* and the mouse (2, 4), suggesting that analysis of the effects of additional L. plantarum strains on *Drosophila* life span would be informative. The second priority is a direct comparison of early-life and late-life effects of L. plantarum on *Drosophila*. There is the fascinating possibility that the L. plantarum strains which reduce *Drosophila* life span, as reported by Fast et al. (1), have the positive early-life effect of accelerated larval growth rates reported by other researchers. Such a result would suggest that L. plantarum (or some strains of this species) may amplify the early- and late-life history tradeoff of their animal host, i.e., promote allocation to growth in early life, even though this reduces the capacity for somatic maintenance later in life (5). These unanswered questions illustrate a more general priority: that a comprehensive understanding of the effects of gut microbes on host health requires the integration of microbial impacts across the different life stages of the host, including juvenile growth, adult fecundity, and life span, ideally on diets of different nutritional quality. This is a substantial research effort, but the short life span and suitability of *Drosophila* for complex experimental designs make this system superbly amenable to this type of analysis.

The second unexpected result of Fast et al. (1) is the mechanistic basis of the reduced life span of *Drosophila* monoassociated with L. plantarum. This requires consideration of the cellular organization of the *Drosophila* gut. As in other animals, the gut is lined by a cellular epithelium which, in adult *Drosophila*, comprises a single layer of differentiated cells. Importantly, these cells are lost continuously by delamination, such that the maintenance of the gut depends on the sustained division of stem cells followed by differentiation of one of the daughter cells into a mature epithelial cell. Complete turnover of the midgut epithelium in *Drosophila* takes approximately 7 days, and the turnover rate is reduced in axenic flies (6). Previous studies have shown that L. plantarum promotes epithelial turnover by stimulating host production of reactive oxygen species (ROS) (7). Bacterium-induced stem cell proliferation has also been invoked to account for the several reports of reduced life span of conventional flies (i.e., *Drosophila* bearing gut microbes) relative to axenic flies, with evidence that heightened stem cell activity is associated with perturbations to the pattern of cellular differentiation and loss of barrier function of the gut epithelium in the aging fly (6, 8, 9).

Surprisingly, the data of Fast et al. (1) do not fit with the expectation that the reduced life span of *Drosophila* monocolonized with L. plantarum is associated with increased stem cell proliferation. Division of stem cells in the *Drosophila* midgut is activated by epidermal growth factor (EGF), with elevated expression of the genes *spitz* and *rhomboid*, coding for EGF and EGF-activating peptidase, respectively (10), but the flies monoassociated with L. plantarum displayed the reverse gene expression response, predictive of reduced stem cell division rates. This interpretation was confirmed by a second set of experiments in which Fast et al. (1) applied MARCM (mosaic analysis with a repressible cell marker) to label with green fluorescent protein (GFP) the progeny of cells from single *Drosophila* stem cells. Both the number of GFP-labeled clones and the number of cells per clone were significantly lower in the flies monoassociated with L. plantarum than in axenic flies. (Consistent with predictions from previous research [10], these indices were higher in conventional flies than axenic flies.) Parallel light and electron microscopy studies confirmed the reduction of proliferative cells and revealed large-scale perturbation to the epithelium of the posterior midgut in the flies monoassociated with L. plantarum.

Although unexpected, the results of Fast et al. (1) are part of a recurring pattern of inconsistency between different studies. The gut microbiota may variously increase, decrease, or have no significant effect on *Drosophila* life span (8, 9, 11, 12), and it is now evident that shortened life span may be associated with overactivation or depletion of stem cell populations. We tend to explain these inconsistencies in terms of context dependence: it all depends on diet, bacterial strain, among-microbe interactions, host genotype, etc., and, likely, higher-order interactions among these different factors.

How are we to make sense of this complexity and inconsistency? I believe that the solution is 2-fold. The first is to minimize the sources of uncontrolled variation, especially to work with standardized microbiota, diet formulations, and culture conditions that are readily transferrable between laboratories. The second and complementary approach is to harness the diversity of experimental outcome as a tool to understand mechanism. One possible way forward arises from the observation of Fast et al. (1) that the life span of *Drosophila* declines with increasing abundance of L. plantarum relative to a second gut bacterium, A. pasteurianus. These data raise the possibility that the impact of L. plantarum on the host gut epithelium varies with bacterial load and may be influenced by interactions with other bacteria. There is the opportunity to investigate whether L. plantarum at low density stimulates stem cell proliferation but suppresses proliferation at high density and to test the contribution of bacterium-induced ROS and other immune effectors to these divergent effects of L. plantarum. Comparisons of monoassociations with two-member and more complex standardized communities (13) may additionally reveal the processes that shape gut epithelial function and life span in conventional flies.

In summary, the article of Fast et al. (1) provides an important contribution toward a molecular understanding of the interactions between the bacterial cells and midgut that shape the patterns of stem cell proliferation and differentiation. Sustained research on the association between *Drosophila* and L. plantarum will undoubtedly continue to contribute to our fundamental understanding of the interactions between *Lactobacillus* and animals and support the role of *Drosophila* as an emerging model for probiotic science.
